# Farnesoid X Receptor Activation in Brain Alters Brown Adipose Tissue Function via the Sympathetic System

**DOI:** 10.3389/fnmol.2021.808603

**Published:** 2022-01-04

**Authors:** Benjamin Deckmyn, Dorothée Domenger, Chloé Blondel, Sarah Ducastel, Emilie Nicolas, Emilie Dorchies, Emilie Caron, Julie Charton, Emmanuelle Vallez, Benoit Deprez, Jean-Sébastien Annicotte, Sophie Lestavel, Anne Tailleux, Christophe Magnan, Bart Staels, Kadiombo Bantubungi

**Affiliations:** ^1^EGID, U1011, University of Lille, Lille, France; ^2^Inserm, U1011, Lille, France; ^3^CHU Lille, Lille, France; ^4^Institut Pasteur de Lille, Lille, France; ^5^Laboratory of Lille Catholic Hospitals, Medical Biology Department, Lille Catholic University, Lille, France; ^6^Inserm UMR-S 1172, Lille, France; ^7^Inserm U1177, Lille, France; ^8^Drugs and Molecules for Living Systems, U1177, University of Lille, Lille, France; ^9^CNRS UMR 8199, Lille, France; ^10^CNRS UMR 8251, Paris, France; ^11^University Paris Diderot, Paris, France

**Keywords:** FXR, brain, hypothalamus, energy homeostasis, brown adipose tissue

## Abstract

The nuclear bile acid (BA) receptor farnesoid X receptor (FXR) is a major regulator of metabolic/energy homeostasis in peripheral organs. Indeed, enterohepatic-expressed FXR controls metabolic processes (BA, glucose and lipid metabolism, fat mass, body weight). The central nervous system (CNS) regulates energy homeostasis in close interaction with peripheral organs. While FXR has been reported to be expressed in the brain, its function has not been studied so far. We studied the role of FXR in brain control of energy homeostasis by treating wild-type and FXR-deficient mice by intracerebroventricular (ICV) injection with the reference FXR agonist GW4064. Here we show that pharmacological activation of brain FXR modifies energy homeostasis by affecting brown adipose tissue (BAT) function. Brain FXR activation decreases the rate-limiting enzyme in catecholamine synthesis, tyrosine hydroxylase (TH), and consequently the sympathetic tone. FXR activation acts by inhibiting hypothalamic PKA-CREB induction of TH expression. These findings identify a function of brain FXR in the control of energy homeostasis and shed new light on the complex control of energy homeostasis by BA through FXR.

## Introduction

Proper energy homeostasis is crucial to maintain health and avoid the development of metabolic disorders such as obesity, dyslipidemia and type 2 diabetes. The essential role of the CNS in the regulation of energy homeostasis is now well documented (Bantubungi et al., 2012; Kim et al., 2018). The CNS closely interacts with peripheral organs to gather information on its energy state and provides, in turn, signals to adapt biological responses. Among the different brain regions controlling energy homeostasis, the hypothalamus is the major center of convergence and integration of nutrient/hormonal signals and environmental cues, particularly through two types of neurons, pro-opio-melanocortin (POMC)-producing neurons and agouti-related protein (AgRP)-producing neurons of the arcuate nucleus of hypothalamus (ARH). Moreover, the CNS communicates with peripheral organs, such as the liver, intestine and adipose tissue through the peripheral nervous system.

The Farnesoid X Receptor (FXR) belongs to the nuclear receptor superfamily of ligand-regulated transcription factors (Lefebvre et al., 2009). FXR is highly expressed in the liver and intestine, where it regulates the expression of target genes to control BA, glucose and lipid metabolisms (Lefebvre et al., 2009; Chávez-Talavera et al., 2017). Although expressed at much lower levels, a role for FXR has also been suggested in peripheral organs such as pancreas and adipose tissue (Cariou et al., 2007; Abdelkarim et al., 2010; Popescu et al., 2010; Seyer et al., 2013). FXR has also been involved in the control of energy homeostasis as exemplified by the reduction of body weight and adipose tissue mass in FXR-KO mice. In accordance, FXR deficiency was shown to protect from excessive weight gain in genetic and diet-induced (DIO) obesity models (Prawitt et al., 2011; Zhang et al., 2012), while FXR activation, by peripheral administration of GW4064, potentiated body weight gain and glucose intolerance in DIO mice (Watanabe et al., 2011). All studies investigating the metabolic control by FXR have so far exclusively focused on its action in peripheral organs (Lefebvre et al., 2009; Chávez-Talavera et al., 2017). While FXR reportedly is expressed in the brain (Gofflot et al., 2007; Huang et al., 2016), its function remains ill-defined. So far, studies evaluating BA-FXR signaling in the CNS mainly focused on its potential role in neurodegenerative conditions, such as Alzheimer’s disease (Lo et al., 2013; Bell et al., 2018; McMillin et al., 2018), Parkinson’s disease (Castro-Caldas et al., 2012; Abdelkader et al., 2016; Moreira et al., 2017; Rosa et al., 2018), Huntington’s disease (Keene et al., 2001, 2002) as well as amyotrophic lateral sclerosis (Vaz et al., 2015; Elia et al., 2016). In these models of neurodegeneration, BA-FXR activation appears rather protective by acting on its pathophysiological mechanisms. Huang et al. showed alterations in depressive-like and anxiety-related behaviors in FXR-deficient mice, linked to an alteration of neurotransmitter homeostasis in different brain regions. The authors assumed that these effects are potentially mediated by changes in plasma and brain BA pools (Huang et al., 2015). Although the brain is well-recognized as an important regulator of peripheral homeostasis, whether brain FXR plays a role in the regulation of metabolism in peripheral organs remained unexplored. Given the importance of the CNS in the control of energy homeostasis and the role of FXR as a metabolic/energy sensor in peripheral organs, we investigated the role of brain FXR in the control of energy homeostasis. Here, we identify an unexpected novel function of brain FXR in its ability to control BAT function via a mechanism involving modification of hypothalamic PKA-CREB signaling and, subsequently, the sympathetic tone.

## Materials and Methods

### Animals

Animal experiments were approved by the Institutional Committee for animal use and care. The ethical committee of the University of Lille approved all protocols (APAFIS#11237-20170911185145v2, APAFIS#13331-2017091915214567v17). Male wild-type mice (C57BL/6J), male FXR-deficient mice [FXR-KO, provided by Sinal et al. (2000)] and their littermates (FXR-WT), on the C57BL/6J genetic background (Charles River), 16–19 weeks old, were housed under a 12 h/12 h light/darkness cycle in temperature (21.5°C) and humidity controlled rooms, in a specific pathogen-free environment. Standard diet (A04, Safe) and water were available *ad libitum* except during the cold-exposure experiment. Depending on the protocols, mice were either placed in metabolic cages (TSE systems, Hamburg, Germany) or standard cages and scarified in the fed status. All experiments were performed with minimum of 5 animals per group. The precise number of animals per group is mentioned in figure legends.

### Intracerebroventricular Cannulations

Mice were randomized based on body weight, anesthetized using a mixture of ketamine (75 mg/kg)/xylazine (10 mg/kg), and stereotactically equipped with a cannula targeting the lateral ventricle of the brain (AP: + 0.24 mm, ML: + 1 mm). The cannula was secured on the skull with dental ciment.

### Farnesoid X Receptor Agonist Treatments

For intracerebroventricular (ICV) treatment, the synthetic FXR specific agonists GW4064 (3-(2,6-Dichlorophenyl)-4-(3′-carboxy-2-chlorostilben-4-yl)-oxymethyl-5-isopropylisoxazole, Tocris, 2473/50, purity > 97%) and tropifexor (LJN452, Clinisciences) were dissolved in 100% DMSO and injected in a volume of 1 μl, 0.25 μl/min. In the chronic injection experiment, one ICV injection was administered every day (at the end of the day just before light turn off) for 6 days (GW4064 at the dose of 0.9 mM or tropifexor at the dose of 0.2 mM (doses selected after dose-response experiments in metabolic cages) or vehicle (100% DMSO) and mice were sacrificed the day after the last injection. Peripheral blood concentration of GW4064 was determined by high-performance liquid chromatography combined with mass spectrometry (LC-MS/MS Acquity I-Class – Xevo TQD Waters).

### Mouse Monitoring in Metabolic Cages

Wild-type mice were individually placed in metabolic cages (TSE systems, Hamburg, Germany). Energy parameters were recorded throughout the experiment: food intake (in grams, by minute and cumulative over 48 h), locomotor activity (number of cage crossings, number of straightenings), VO_2_ and CO_2_ consumption (in ml/h/kg lean mass) was measured and energy expenditure calculated (Weir formula: EE = (3.94 × VO_2_ + 1.106 × VCO_2_)/1000 in kcal/h/kg lean mass) and the Respiratory Exchange Ratio (RER).

### Sympathetic Nerve Activity Recording

Mice were anesthetized using a mixture of ketamine (75 mg/kg)/xylazine (10 mg/kg), and the carotid artery was exposed. The sympathetic nerve filament was dissected free of underlying tissues on a distance of 1 cm until the superior cervical ganglion. The nerve was covered with paraffin oil and placed on a pair of recording silver electrodes (0.6 mm diameter) connected to a high-impedance probe, action potentials were saved after initial amplification through a low-noise amplifier (BIO amplifier, ADInstrument, Paris, France). Unipolar nerve activity was recorded continuously for 15 min. Data were digitized with PowerLab/4sp digitizer (ADInstrument, Paris, France). Signals were amplified 105×, filtered using low/high-frequency cut-offs of 100 and 1,000 Hz, and monitored using the Chart 4 computer program (ADInstrument, Paris, France).

### Cold-Exposure Experiment

C57BL/6J mice were individually housed at 4°C during 8 h without access to food and water during the cold-exposure period only. The rectal temperature was monitored using a rectal thermoprobe at the end of cold-exposure.

### RNA Extraction, cDNA Synthesis and Quantitative Real-Time PCR

PVH, ARH, liver, BAT and eWAT were dissected and frozen in liquid N_2_. Total RNA was isolated using the RNeasy Lipid Tissue Mini Kit (Qiagen). Retrotranscription reactions were performed using the cDNA Reverse Transcription High Capacity Kit (Applied Biosystem). QPCR reactions were performed using Brilliant Sybr Green II QPCR Master Mix kit on the Stratagene MX3000P device (Agilent Technologies) or TaqMan Multiplex Master Mix (Applied Biosystem) on the Applied Biosystems 7500 Real Time PCR device. mRNA levels were normalized to a control gene (cyclophillin for Sybr green, 18S for Taqman) whose expressions are not influenced by the experimental conditions. Supplementary Table 1 details the primer sequences used for real-time PCR.

### Western Blot Analysis

Hypothalamus, BAT and liver were dissected and frozen in liquid N_2_. Tissues were homogenized in 1 ml buffer (Trizmabase/sucrose) and sonicated. 20 μg of protein in LDS sample buffer were loaded per lane, separated with NuPage 4–12% Bis-Tris Protein Gels (Thermofischer, NP0335BOX) and transferred to nitrocellulose membrane iBlot 2 transfert stacks (Thermofischer, IB23001). Membranes were immunoblotted at 4°C overnight with antibodies against UCP1 (Abcam, AB10983, rabbit polyclonal, IgG; 1/500), Tyrosine Hydroxylase (Merck, MAB5280, mouse monoclonal, IgG; 1/500), FXR (Abcam Perseus, PP-A9033A-00, mouse monoclonal, IgG; 1/500), PKARII (BD Biosciences, 610626, mouse monoclonal, IgG; 1/250), PPKARII (BD Biosciences, 612550, mouse monoclonal, IgG; 1/250), HSP 90α/β (H-114) (Santa cruz biotechnology, sc-7947, rabbit polyclonal, IgG; 1/1000) and βActine (Sigma, A5441, mouse monoclonal, IgG; 1/1000). All antibodies were diluted in Tris buffer saline (TBS) supplemented with Tween 0.01% and milk powder 5%. The secondary antibodies used are goat anti mouse (Sigma A4416, IgG) or anti-rabbit (Sigma A0545, IgG), diluted in TBS supplemented with Tween 0.01% and milk powder 5%. The incubations are performed at room temperature 2 h. Results are represented in the form of boxes for illustration purposes. All samples of an experiment were processed on the same western blot. When different gels were necessary, one or more common samples were run on each gel to allow subsequent normalization of the results.

### Immunohistochemistry Analysis

Mice were anesthetized using a mixture of ketamine (75 mg/kg)/xylazine (10 mg/kg), followed by intracardiac perfusion using a solution of saline (NaCl 0.9%, 20 ml) and subsequently a solution of 4% PFA. BAT was fixed by immersion at 4°C in 4% PFA, dehydrated, cleared and embedded in paraffin. Paraffin sections (5 μm thick) were stained with hematoxylin and eosin. Brains were post-fixed for 16 h at 4°C in 4% PFA. Cryoprotection of brains was performed by successive 24 h baths of PBS-sucrose buffer (10, 20, 30%) at 4°C. Brains were included in Tissue Freezing Medium (Jung) before being frozen in isopentane cooled to −55°C with liquid nitrogen. Blocks were stored at −80°C. Brains were cut at 18 μm using a Leica cryostat (CM3050) and placed on Superfrost Plus slides (Thermofischer) for FXR (Abcam, AB28676, rabbit polyclonal, IgG; 1/100), αMSH (Millipore AB5087, sheep polyclonal, IgG; 1/10000), HuD/C (Thermofischer, A-21271, mouse monoclonal, IgG; 1/500) staining. Free-floating slices of 40μm were prepared for p-CREB immunohistochemistry. FXR-NPY co-staining was performed on mouse brain sections whose expression of the fluorescent GFP protein is under the control of the NPY promoter, not requiring double labeling. For FXR/αMSH and FXR/HuD/C co-staining, slides were incubated for 16 h at 4°C with FXR antibody diluted at 1/100 (PBS 0.01M + 1% blocking reagent), followed by an incubation for 2 h at room temperature with the secondary anti-rabbit antibody coupled to HRP (Thermofischer, B40922, goat polyclonal, IgG) diluted at 1/100 (PBS 0.01M + 1% blocking reagent). Next, the slides were incubated in biotinylated tyramide diluted at 1/100 (amplification buffer, Thermofischer, B40922 + H2O2 0.0015%) for 5 min at room temperature. The second step was an incubation for 16 h at 4°C in the presence of αMSH antibody diluted at 1/10000 (PBS 0.01M + 0.1% X-100 newt + 1% donkey serum) or HuD/C antibody diluted at 1/500 (PBS 0.01M + 0.1% X-100 newt + 1% goat serum). Then, slides were incubated for 2 h at room temperature with the secondary antibody coupled to a fluorochrome emitting at 555 nm (for MSH) (Molecular probe, A-21432, donkey polyclonal, IgG) or 568 nm (for HuD/C) (Invitrogen, A-31570, donkey polyclonal, IgG) diluted at 1/200 (PBS 0.01M + 0.1% newt X-100). Lastly, labeling was performed using a 1/1000 diluted Hoechst solution in 0.01M PBS (Invitrogen^®^, Hoechst33258) and slides were mounted with lamellae (Dako^®^ Fluorescent Mounting Medium).

For Free-floating immunohistochemistry, brain sections were placed in 12-wells culture plates and incubated for 16 h at 4°C with PCREB (Ser133) (87G3) (Cell Signaling, 9198S, rabbit polyclonal, IgG, goat polyclonal, IgG) antibody diluted at 1/1000 (PBS 0.01M + 0.2% triton X-100 + 1% goat serum). Then, sections were incubated for 2 h at room temperature with the secondary antibody coupled to a fluorochrome emitting at 488 nm (Molecular probe, A11008) diluted at 1/1000 (PBS 0.01M). Finally, labeling was performed using a 1/5000 diluted Hoechst solution and slides mounted on Superfrost Plus slides.

The image acquisition was performed using a confocal microscope (LSM710, Zeiss). Quantification and analysis of signals were done using Imaris software. For quantitative analysis of p-CREB in ARH neurons, a total of 16951 neurons of control mice and 12704 neurons of treated mice were counted in ARH slices of independent control mice (*n* = 8) and GW4064-treated mice (*n* = 7), and the amount of pCREB was classified as either low, moderate or high based on the level of fluorescence intensity given by Imaris software.

### Hybridation *in situ*

Fluorescent *in situ* hybridization was performed using RNAscope^®^ Multiplex Fluorescent Reagent Kit 2.0 according to the manufacturer’s instructions (Advanced Cell Diagnostics). Briefly, brain sections (20 μm) were fixed in 4% paraformaldehyde for 1 h at 4°C and dehydrated through graded ethanol solutions (50, 70, and 100%) for 5 min each. Sections were treated by hydrogen peroxyde reagent at room temperature for 10 min and then hybridized with probes at 40°C for 2 h in a humidified oven. The *NR1H4* probe (FXR; Cat# 484491), *POMC* probe (Cat# 314081-C3) and the *NPY* probe (Cat# 313321-C2) were used. After hybridization, brain sections were sequentially applied with a series of probe signal amplification steps (Opal 520, 570 and 690), rinsed with ACD wash buffer twice for 2 min between each step. Lastly, nuclear labeling was performed using a 1/1000 diluted Hoechst solution in 0.01M PBS (Invitrogen^®^, Hoechst33258) and slides were mounted with lamellae (Dako^®^ Fluorescent Mounting Medium).

### Statistical Analyses

All values are reported as means ± SEM. Data were analyzed using the unpaired Student’s *t*, two-way ANOVA or X_2_ tests, using the Prism software (GraphPad, United States). Significance was set at *P* < 0.05 for all experiments.

### Data Statement

The data sets generated during and/or analyzed during the current study are available from the corresponding author upon reasonable request.

## Results

### Intracerebral Treatment With GW4064 Activates Brain Farnesoid X Receptor and Modifies Energy Homeostasis, Brown Adipose Tissue Function and the Sympathetic Tone

**FIGURE 1:**
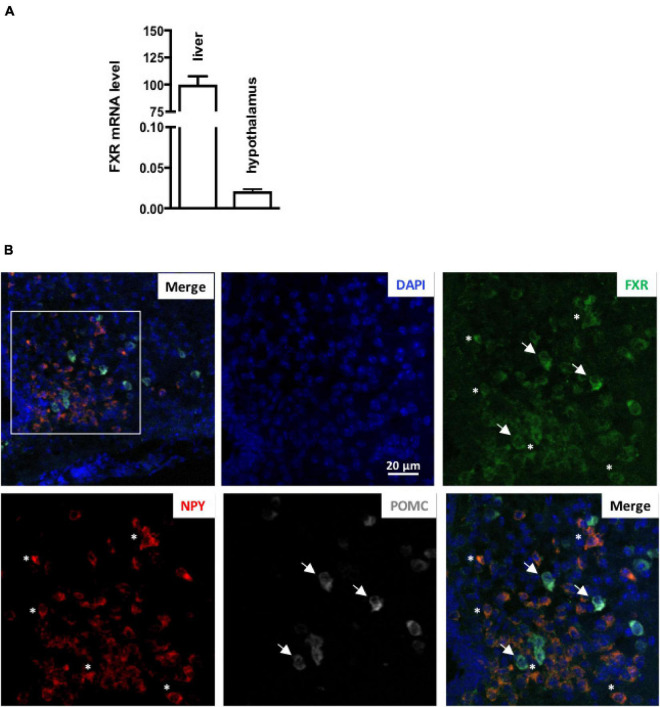
FXR mRNA is expressed in hypothalamus. **(A)** *Fxr* mRNA expression in liver and hypothalamus by qPCR. The values are normalized to cyclophilin. Results are expressed by comparing the expression of FXR in the liver, whose expression level has been arbitrarily set at 100%. **(B)** Representative images of RNAscope staining of FXR with NPY and POMC in ARH. The nuclear staining (blue) was performed with Hoechst solution. 3V, third ventricle; ARH, arcuate nucleus of hypothalamus; ME, median eminence.

**FIGURE 2:**
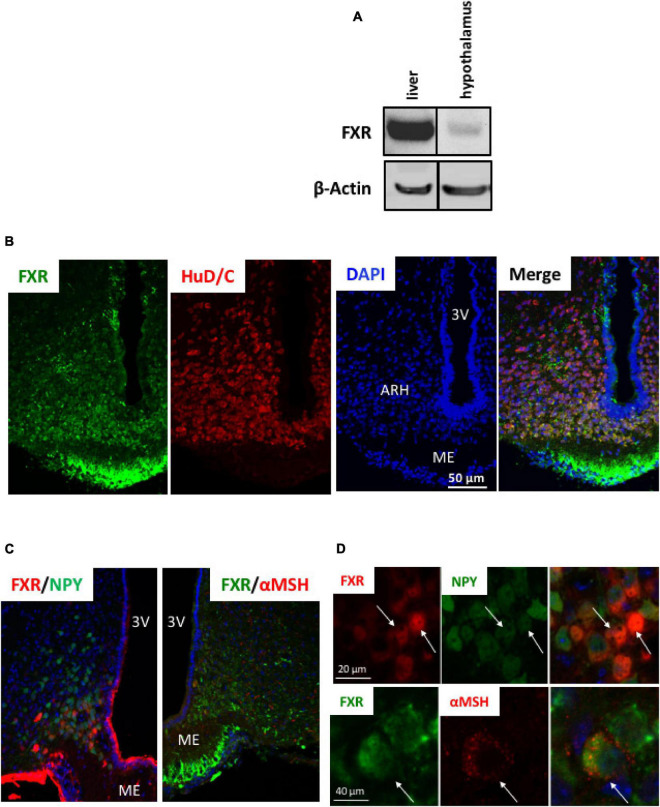
FXR protein is expressed in hypothalamus. **(A)** FXR protein expression in liver and hypothalamus by Western blot. **(B–D)** Representative images of co-immunostaining of FXR with HuD/C **(B)** or NPY or αMSH **(C)** in ARH. **(D)** Higher magnification of panel **(D)**. The nuclear staining (blue) was performed with Hoechst solution. 3V, third ventricle; ARH, arcuate nucleus of hypothalamus; ME, median eminence.

**FIGURE 3:**
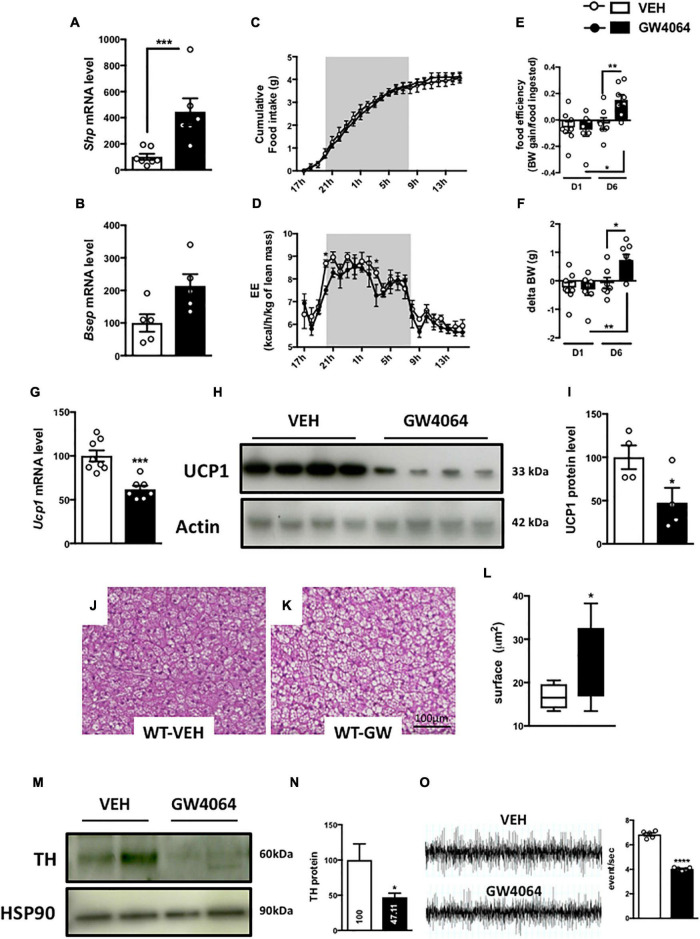
Intracerebral treatment with GW4064 activates brain FXR and modifies energy homeostasis, BAT function and the sympathetic tone. **(A,B)** *Shp* and *Bsep* mRNA expression in ARH by q-PCR. The values are normalized to cyclophilin. **(C)** Cumulative food intake and **(D)** energy expenditure (EE) were measured in metabolic cages. **(E)** Food efficiency was calculated by ratio of body weight and food intake after one day and 6 days of treatment by GW4064. **(F)** Body weight gain was measured after one day and 6 days of treatment. **(G)** *Ucp1* mRNA expression in BAT by q-PCR. The values are normalized to cyclophilin. **(H)** UCP1 protein expression in BAT. **(I)** The bar graph is the quantification of UCP1 western blots in panel **(H)**. **(J,K)** Representative images of histological hematoxylin and eosin (H&E) staining of BAT. **(L)** Quantification of lipid droplet surface. **(M)** TH protein expression in BAT. **(N)** The bar graph is the quantification of TH western blots in panel **(M)**. **(O)** Representative recordings of sympathetic nerve activity from VEH and GW4064 treated mice and histogram of sympathetic nerve activity. Data are mean ± SEM. *P < 0.05, **P < 0.01, ***P < 0.001, ****P < 0.0001, Unpaired Student’s *t* test or Two-Way ANOVA followed by Tukey *post hoc*. Vehicle group is indicated as open circles/bars, GW4064 group as black circles/bars.

**FIGURE 4:**
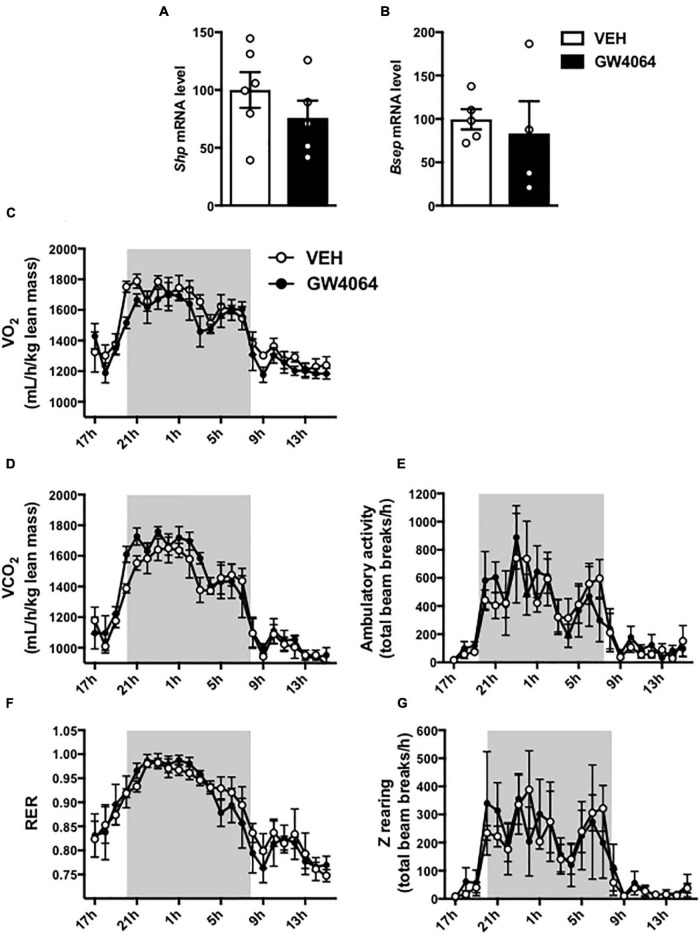
Effect of 6-days cerebral treatment with GW4064 on FXR target gene expression in PVH and energy homeostasis. **(A,B)** *Shp* and *Bsep* mRNA expression in PVH by q-PCR. The values are normalized to cyclophilin. **(C)** 24 h-oxygen consumption (VO_2_). **(D)** 24 h-carbon dioxide consumption. **(E)** 24 h-spontaneous locomotor activity (total beam breaks/hour). **(F)** 24 h-respiratory exchange ratio (RER). **(G)** 24 h-spontaneous Z-rearing (total beam breaks/hour). Data are mean ± SEM. Unpaired Student’s *t* test or Two-Way ANOVA followed by Tukey *post hoc*. Vehicle group is indicated as open circles/bars, GW4064 group as black circles/bars.

To assess if brain FXR contributes to the regulation of energy homeostasis, we first fine-mapped the exact localization of FXR expression in the hypothalamus at mRNA and protein levels (Gofflot et al., 2007; Huang et al., 2016; Figures 1, 2). FXR expression was observed in neurons of the arcuate nucleus (ARH) (Figures 1B, 2B), expressed by both alpha-Melanocyte-Stimulating Hormone and Neuropeptide Y (αMSH and NPY) neurons of the ARH (Figures 1B, 2C,D), which regulate energy homeostasis (Sánchez-Lasheras et al., 2010). To determine whether FXR plays a role in the brain control of energy homeostasis, we treated mice by ICV injection with the reference FXR agonist GW4064 (Maloney et al., 2000). Given the absence of precise data in the literature concerning the bioavailability and pharmacokinetics of GW4064 in the brain, we empirically determined the active dose by performing an effect/dose experiment in metabolic cages. Chow fed lean mice in metabolic cages received increasing doses of GW4064 by ICV administration and modification of metabolic parameters as well as FXR target genes expression were monitored. A dose of 0.9 mM; 522 ng/μl; 1 μl injected, of GW4064, was found to modify metabolic parameters and to increase the expression of established FXR target genes in the hypothalamus, indicative of a pharmacological activity and a cerebral activation of FXR. Indeed, mRNA levels of the FXR target genes *Small Heterodimer Partner* and *Bile Salt Export Pump* (*Shp* and *Bsep*) were induced in the ARH upon GW4064 treatment (Figures 3A,B), but not in the paraventricular nucleus of the hypothalamus (PVH) (Figures 4A,B), indicating specific activation of FXR in the ARH. No differences in respiratory exchange rate (RER), ambulatory activity, Z rearing (Figures 4E–G) nor food intake (Figure 3C) were observed between GW4064 versus vehicle treated mice. However, GW4064 treatment slightly affected VO_2_, VCO_2_ (Figures 4C,D) and weakly but significantly decreased energy expenditure (EE) in the first part of the dark phase (Figure 3D). Moreover, after 6 days of GW4064 treatment, food efficiency was significantly enhanced along with an increased body weight gain, suggesting a positive energy balance (Figures 3E,F).

Because energy homeostasis is a dynamic balance between food intake and energy expenditure (Hall et al., 2012) (including physical activity, basal metabolism and thermogenesis), we next assessed whether brain FXR activation modified basal metabolism or thermogenesis. mRNA levels of genes involved in several pathways related to basal metabolism and thermogenesis were then measured in peripheral organs: liver, epididymal adipose tissue (eWAT) and BAT (Cannon, 2004). ICV treatment with GW4064 did not modify the expression of glucose nor lipid metabolism genes in liver nor in eWAT. Surprisingly, a decrease of *Uncoupling Protein 1* (*Ucp1*) gene and protein expression (Figures 3G–I), a marker of BAT activity (Zhang and Bi, 2015), was found, suggesting that BAT function may be impaired upon CNS FXR activation. Moreover, a decrease of *Vascular endothelial growth factor* (*Vegf*) gene expression (Supplementary Figure 1), a marker of vascularization and an enlargement of brown adipocytes (Figures 3J–L) were found upon GW4064 treatment, suggesting a remodeling of BAT (Shimizu et al., 2014). These effects of CNS FXR activation on BAT functions were not due to systemic leakage of the compound or the activation of peripheral FXR, since GW4064 could not be detected in plasma after ICV GW4064 injection using a highly sensitive analytical system [C < 10 nM(LOQ)] nor were any of the classical FXR target genes induced in livers of these mice.

To understand the action mechanism of CNS FXR on energy expenditure and BAT function, the brain-BAT axis was next investigated by assessing the impact of FXR on the adrenergic tone exercised by the peripheral nervous system. Indeed, BAT contains peripheral sympathetic fibers expressing Tyrosine Hydroxylase (TH) (Razzoli et al., 2016), the rate-limiting enzyme in catecholamine synthesis. These TH-positive fibers release noradrenaline which through β-3 adrenergic receptors induces intracellular signaling increasing UCP1 expression in BAT (Harms and Seale, 2013). Interestingly, in mice ICV treated for 6 days with GW4064, TH expression in afferent BAT adrenergic neurons significantly decreased (Figures 3M,N). Moreover, the activity of the sympathetic nervous system was significantly decreased in mice treated ICV with GW4064 as compared to vehicle controls (Figure 3O). Thus, CNS FXR activation by GW4064 impairs the sympathetic tone in BAT.

**FIGURE 5:**
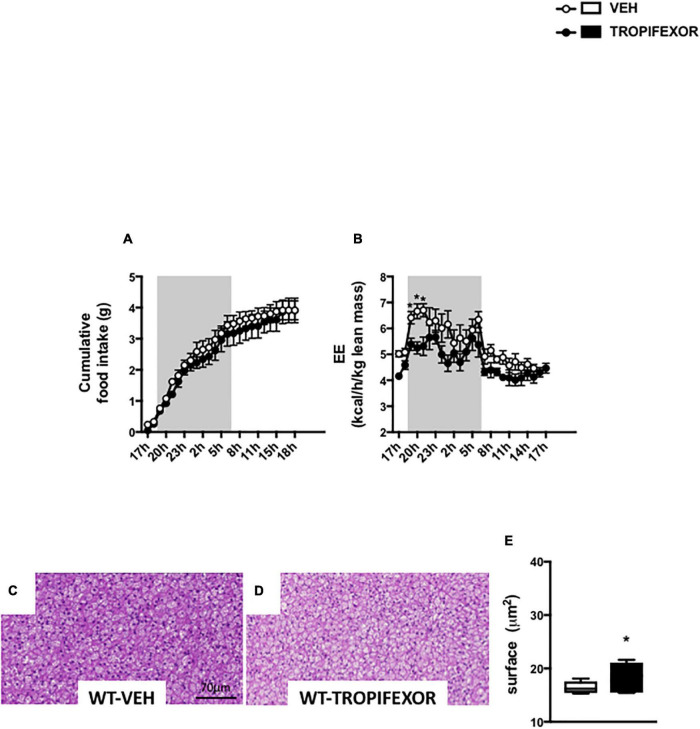
Intracerebral treatment with tropifexor modifies energy homeostasis and BAT function. **(A)** Cumulative food intake and **(B)** energy expenditure (EE) were measured in metabolic cages. **(C,D)** Representative images of histological hematoxylin and eosin (H&E) staining of BAT. **(E)** Quantification of lipid droplet surface. Data are mean ± SEM. *P < 0.05, Two-Way ANOVA followed by Tukey *post hoc*. Vehicle group is indicated as open circles/bars, GW4064 group as black circles/bars.

To ascertain the engagement of brain FXR on energy homeostasis and BAT, additional experiments were performed using a different synthetic FXR agonist, tropifexor, which is currently undergoing phase 2 human clinical trials in NASH and PBC (Tully et al., 2017). Noteworthy, ICV treatment with tropifexor also resulted in a decrease of EE without impacting food intake (Figures 5A,B). Moreover, icv treatment with tropifexor also increased BAT lipid droplet size (Figures 5C–E), indicating that CNS FXR activation affects the BAT remodeling.

### Intracerebral GW4064 Treatment Lowers Brown Adipose Tissue Activity and Rectal Temperature

**FIGURE 6:**
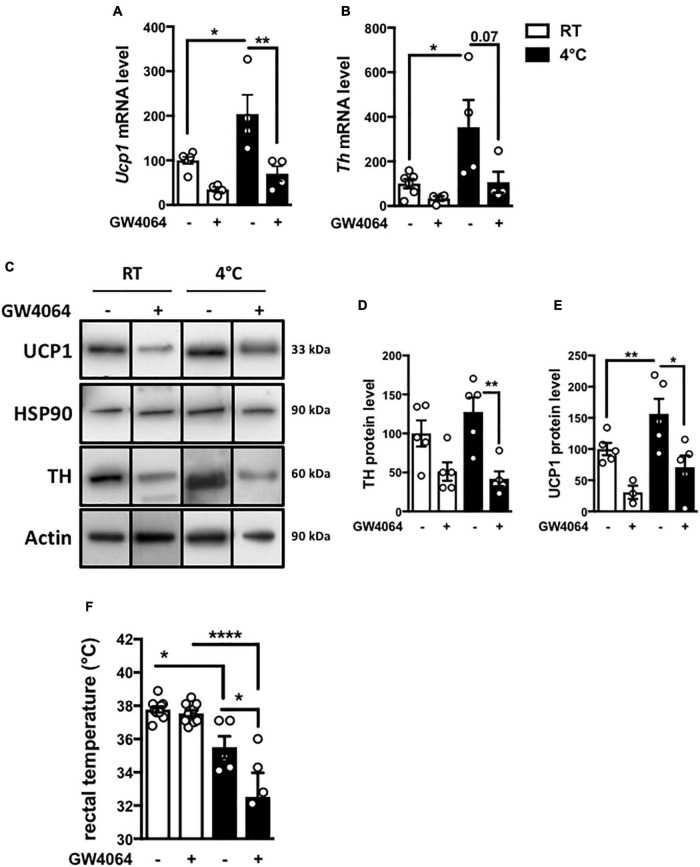
Intracerebral treatment with GW4064 functionally impacts the BAT and rectal temperature. **(A)** *Ucp1* mRNA expression in BAT of mice placed at room temperature (23°C) or 4°C. **(B)** *Th* mRNA expression in BAT of mice placed at room temperature (23°C) or 4°C. The values are normalized to cyclophilin or 18S. **(C)** UCP1 and TH protein expression in BAT of mice placed at room temperature (23°C) or 4°C. Results are represented in the form of boxes for illustration purposes. For an experiment, all samples are processed in the same western blot. If different gels were used if the number of wells was insufficient, we took the precaution of introducing one or more common samples within each gel to standardize the results. **(D)** The bar graphs are the quantification of UCP1 western blots in panel **(C)**. **(E)** The bar graph is the quantification of TH western blots in panel **(C)**. **(F)** Rectal temperature was measured at the end of 23°C- or 4 h-cold exposure. Data are mean ± SEM. *P < 0.05, **P < 0.01, ****P < 0.0001, Two-Way ANOVA followed by Tukey *post hoc*. 23°C group is indicated as open bars, 4°C group as black bars.

To determine whether central FXR activation functionally impacts on BAT function, the effect of ICV GW4064 pretreatment on adaptive thermogenesis was studied by subjecting mice to 8 h cold exposure. As expected, cold exposure induced *Ucp1* and *Th* mRNA and protein levels in vehicle-treated mice (Figures 6A–E). Surprisingly, ICV GW4064 pretreatment severely blunted this induction (Figures 6A–E). Moreover, rectal temperature was significantly lower in mice pretreated ICV with GW4064 as compared to vehicle when placed at 4°C (Figure 6F). Cold exposure is a major stimulus leading to increased metabolic functions in BAT (Harms and Seale, 2013). As expected, mRNA levels of the α 1-, α2- and β3-adrenergic receptors in BAT as well as *Peroxisome Proliferator-Activated Receptor Gamma Coactivator 1-alpha* and *Iodothyronine Deiodinase 2* (*Pgc1a* and *Dio2*) were increased in response to 8 h cold exposure in BAT (Supplementary Figures 2A–E). However, ICV treatment with GW4064 did not modulate the 8 h cold exposure-induced expression of these genes, suggesting a mechanism upstream of adrenergic receptor signaling and excluding a mechanism dependent on thyroid signaling.

### Intracerebral GW4064 Treatment Alters Protein Kinase-c-AMP Response Element-Binding Protein Signaling and Tyrosine Hydroxylase Expression in the Hypothalamus

**FIGURE 7:**
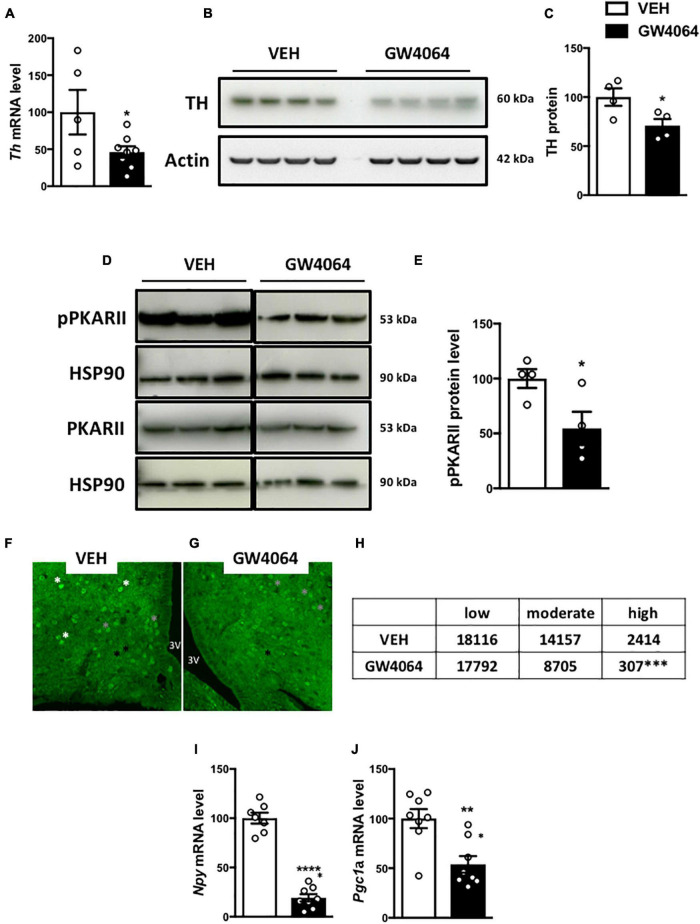
Intracerebral treatment with GW4064 alters PKA-CREB signaling and TH expression in the hypothalamus. **(A)** *Th* mRNA expression in hypothalamus by q-PCR. The values are normalized to 18 s. **(B)** TH protein expression in hypothalamus. **(C)** The bar graphs are the quantification of TH western blots in panel **(B)**. **(D)** pPKARII and PKARII protein expression in hypothalamus. Results are represented in the form of boxes for illustration purposes. For an experiment, all samples are processed in the same western blot. If different gels were used if the number of wells was insufficient, we took the precaution of introducing one or more common samples within each gel to standardize the results. **(E)** The bar graphs are the quantification of pPKARII western blots in panel **(D)**. **(F,G)** Representative images of pCREB immunostaining in ARH of vehicle group **(F)** and GW4064 group **(G)**. **(H)** Quantification of pCREB-positive cells with a low (black asterisk), moderate (gray asterisk), and high staining (white asterisk). **(I,J)** *Npy* and *Pgc1a* mRNA expression in ARH. The values are normalized to cyclophilin. Data are mean ± SEM. *P < 0.05, **P < 0.01, ***P < 0.001, ****P < 0.0001, Unpaired Student’s *t* test. For pCREB quantification, X2 test was performed. Vehicle group is indicated as open bars, GW4064 group as black bars.

To determine how cerebral FXR activation regulates the sympathetic tone in BAT, TH expression was measured in the hypothalamus. TH neurons are located in the PVH of the hypothalamus and project to the brainstem and spinal cord autonomic regulatory centers to integrate sympathetic outflow, for example to BAT (Swanson and Sawchenko, 1983). Interestingly, ICV GW4064 treatment decreased *Th* mRNA and TH protein expression in the hypothalamus (Figures 7A–C). TH expression in the hypothalamus is under control of the transcription factor c-AMP Response Element-Binding protein (CREB) upon its phosphorylation by c-AMP-dependent Protein Kinase (PKA) (Piech-Dumas and Tank, 1999), through interaction with PKARII in the hypothalamus (Yang and McKnight, 2015). Interestingly, PKARII protein phosphorylation was lower in the hypothalamus upon ICV GW4064 treatment (Figures 7D,E), which was associated with a decrease in pCREB immunostaining in the ARH (Figures 7F–H). Moreover, ICV GW4064 treatment decreased mRNA levels of the well-known CREB target genes *Npy* and *Pgc1a* (Herzig et al., 2001; Pandey, 2003) in the ARH (Figures 7I,J). These data demonstrate that pharmacological activation of hypothalamic FXR alters hypothalamic PKA-CREB signaling, hence modulating TH expression in the hypothalamus, and ultimately reducing the sympathetic tone on BAT, thus affecting energy homeostasis.

### Farnesoid X Receptor Mediates the Effects of Intracerebral GW4064 Administration on Brown Adipose Tissue

**FIGURE 8:**
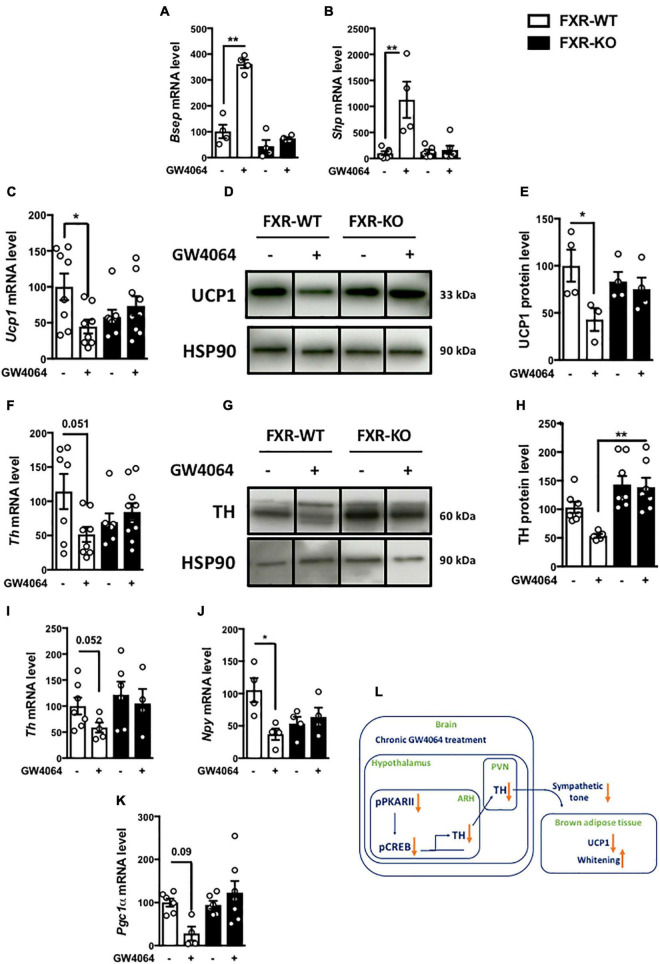
FXR mediates the effects of intracerebral treatment with GW4064 on BAT. **(A)** Besp, **(B)** *Shp* and **(I)** *Th* mRNA expression in hypothalamus of FXR-WT and FXR-KO mice receiving vehicle solution or GW4064 by ICV injection. The values are normalized to cyclophillin or 18 s. **(C)** *Ucp1* mRNA expression in BAT of FXR-WT and FXR-KO mice receiving vehicle solution or GW4064 by ICV injection. The values are normalized to cyclophilin. **(D)** UCP1 protein expression in BAT. **(E)** The bar graphs are the quantification of UCP1 western blots in panel **(D)**. **(F)** *Th* mRNA expression in BAT of FXR-WT and FXR-KO mice receiving vehicle solution or GW4064 by ICV injection. The values are normalized to 18 s. **(G)** TH protein expression in BAT. Results are represented in the form of boxes for illustration purposes. For an experiment, all samples are processed in the same western blot. If different gels were used if the number of wells was insufficient, we took the precaution of introducing one or more common samples within each gel to standardize the results. **(H)** The bar graphs are the quantification of TH western blots in panel **(G)**. **(J,K)** *Npy* and *Pgc1a* mRNA expression in ARH. The values are normalized to cyclophilin. Data are mean ± SEM. *P < 0.05, **P < 0.01, Two-Way ANOVA followed by Tukey *post hoc*. FXR-WT group is indicated as open bars, FXR-KO group as black bars. **(L)** Model: Hypothalamic activation of FXR decreases the activity of PKA-CREB in the hypothalamus, leading to a decrease of hypothalamic TH expression, which disrupts brain-BAT axis.

To ensure the FXR dependence of the response to ICV GW4064, we next investigated the impact of ICV GW4064 in FXR-deficient mice and their littermate controls. The induction of FXR target genes (*Bsep* and *Shp*) in the ARH upon ICV GW4064 treatment in wild-type (FXR-WT) mice was not observed in FXR-deficient (FXR-KO) mice (Figures 8A,B). Moreover, the effect of ICV GW4064 treatment on Ucp1 (Figures 8C–E) and TH (Figures 8F–H) protein expression in BAT observed in FXR-WT mice was not observed in FXR-KO mice. Furthermore, the reduction of hypothalamic *Th*, *Npy* and *Pgc1a* mRNA gene expression observed in FXR-WT mice upon ICV GW4064 treatment was also not observed in FXR-KO mice (Figures 8I–K). These data collectively identify a role of FXR in brain control of energy homeostasis through a brain-BAT axis involving hypothalamic FXR whose activation decreases energy expenditure (Figure 8L).

## Discussion

FXR is a key regulator of entero-hepatic metabolism. Besides its action on BA, lipid and glucose metabolism, FXR regulates insulin sensitivity and GLP1 secretion (Trabelsi et al., 2015; Chávez-Talavera et al., 2017). Moreover, whole body FXR deficiency induces a resistance to diet or genetic induced obesity (Prawitt et al., 2011; Zhang et al., 2012). Interestingly, we found that FXR is expressed in the hypothalamus, a brain area playing a crucial role in the regulation of energy homeostasis (Waterson and Horvath, 2015; Kim et al., 2018). To investigate whether brain FXR activation controls energy homeostasis, brain FXR was pharmacologically activated using the selective FXR agonist GW4064. Our results demonstrate that activation of brain FXR by ICV GW4064 administration leads to histological remodeling of BAT (i.e., increase of lipid droplet size) through hypothalamic regulation of the sympathetic tone. Importantly, these changes promoted by ICV FXR agonist treatment were absent in FXR-KO mice indicating target-selectivity of the compound. In line with previous findings that GW4064 activates the expression of *Shp*, a known FXR target gene, in primary neuronal cultures (Huang et al., 2016), we found that ICV administrated GW4064 also induces its target genes *in vivo* (Figures 3A,B), in a FXR-dependent manner (Figures 8A,B).

Our results showed altered BAT adrenergic activity (decreased TH protein expression), associated with a decrease of sympathetic tone activity, upon ICV GW4064 administration (Figure 3O). The metabolic cage analysis did not allow assessment of possible consequences of the altered autonomic nervous system activity on major cardiovascular functions (heart rate, contractility, arterial pressure). Nevertheless, after 6 days of ICV GW4064 treatment, we observed slight decreases in O_2_ and increases in CO_2_ consumption at night. These observations are in line with a modified sympathetic tone, since disorders of sympathetic control or modulation of adrenergic receptors are known to impact O_2_ and CO_2_ consumption (Narkiewicz et al., 2006; Houssiere et al., 2007; Witte et al., 2008)(Ek and Åblad, 1971; Lang et al., 1989; Billinger et al., 2001; Witte, 2003).

The limited treatment duration (6 days) may explain the relatively mild, albeit significant metabolic changes observed in the metabolic cages. However, the effects on BAT are substantial as demonstrated by the pronounced changes in UCP1 protein expression, BAT histology and response upon cold exposure (Figures 3G–L, 6). Further studies are required to assess the impact of CNS FXR activation on energy metabolism in models of chronically altered energy homeostasis, such as in diet-induced obesity. Such studies are, however, difficult to perform given the invasive nature of the ICV delivery of GW4064.

Even though the ICV administered amounts of GW4064 are many-fold lower than the amounts typically given *per os* or *ip*, to exclude possible effects of peripheral leakage of GW4064 following ICV injection, plasma GW4064 concentrations were measured using a highly sensitive analytical system (high performance liquid chromatography coupled with mass spectrometry). This assay revealed undetectable GW4064 amounts in the plasma of mice treated 6d with ICV GW4064. Moreover, none of the classical FXR target genes were found to be induced in the liver arguing against a systemic leakage of the compound and/or a peripheral activation of FXR (Supplementary Figures 3A,B). Finally, whereas BAT has been shown to express the G-protein coupled membrane BA receptor TGR5, expression of FXR was not detectable by western blot analysis, confirming previous reports (Watanabe et al., 2006). Moreover, GW4064 has no activity on TGR5, ruling out such off-target effects (Maloney et al., 2000). All these data exclude the possibility that the observed alteration of BAT function is due to a peripheral activation of FXR induced by a leakage of GW4064 (from the brain to the periphery).

Peripheral administration of GW4064 has been shown to alter hepatic expression of genes involved in autophagy through mechanisms implicating a cross-talk with CREB (Seok et al., 2014). Our data indicate that activation of FXR in the brain acts through a similar mechanism: inhibition of the PKA-CREB pathway in the ARH leading to a decrease of CREB target gene expression. Although we have shown that central pharmacological activation of FXR alters PKA-CREB signaling, the direct mechanism is unknown and remains to be elucidated. FXR thus appears to control the CREB signaling pathway in different tissues.

While the studies evaluating BA signaling or BA-FXR signaling in the CNS mainly focused on its potential role in neurodegenerative conditions (Keene et al., 2001, 2002; Castro-Caldas et al., 2012; Lo et al., 2013; Vaz et al., 2015; Abdelkader et al., 2016; Elia et al., 2016; Moreira et al., 2017; Bell et al., 2018; McMillin et al., 2018; Rosa et al., 2018), very few papers investigated so far the role of FXR in brain control of energy homeostasis. A recent study reported that brain administration of the BA tauro-lithocholic acid (LCA) promotes fat oxidation and decreases fat mass associated with enhanced fatty acid uptake by BAT and browning of subcutaneous white adipose tissue (Eggink et al., 2018). Since LCA is a specific TGR5 agonist, without activity on FXR, these observations suggest opposite functions of the membrane BA receptor TGR5 and the nuclear receptor FXR. Similar contrasting roles of TGR5 and FXR have been observed previously with respect to the regulation of GLP1 secretion by L cells (Seyer et al., 2013; Trabelsi et al., 2015). In the same line, Castellanos-Jankiewicz’s paper (Castellanos-Jankiewicz et al., 2021) show that the hypothalamic TGR5 activation by this BA mix induces an increased activity of the sympathetic nervous system (SNS) impacting energy expenditure without food intake or body weight changes, while our results demonstrate that hypothalamic activation of FXR induces a decreased activity of the SNS leading to a decreased energy expenditure. These results actually mirror those obtained in our study i.e., hypothalamic activation of FXR decreases activity of the SNS leading to a decreased energy expenditure. Data obtained in other organs, such as the intestine, have already revealed that FXR and TGR5 often exert antagonistic functions (Seyer et al., 2013; Trabelsi et al., 2015). It is likely that temporal differences in response to activators occur for TGR5 and FXR, the former upon activation transmitting its signal rapidly, whereas the former, acting as a transcription factor, allows a slower, more chronic response since this requires modulation of gene expression.

Thus, TGR5 and FXR likely also exert opposite actions on brain control of energy homeostasis to finely control, through the SNS, energy homeostasis and particularly energy expenditure. Overall, this implies that prevention of energy disorders would be achieved by stimulating TGR5 and/or inactivating hypothalamic FXR. Accordingly, Castellanos-Jankiewicz et al. show that the levels of TbMCA, one of the most abundant BA in plasma and in the hypothalamus and a known antagonist of FXR, strongly drop in DIO conditions.

Eggink et al. (2018) reported that ICV GW4064 (10 μM in final concentration in cerebrospinal fluid) did not modulate energy metabolism. The disparity of their results and ours may relate to the used dose, which in the present study was 3× greater than the one used by Eggink et al. Given the absence of precise data in the literature concerning the bioavailability and pharmacokinetics of GW4064 in the brain, it was crucial to perform preceding dose-response experiments *in vivo* to determine the efficient dose. Therefore, we first performed a dose-response experiment with increasing doses of GW4064 by ICV. Similar as Eggink et al., at the dose of 10 μM GW4064, we observed very limited effects of GW4064 on energy metabolism, which became pronounced when the GW4064 dose was increased to 30 μM, both with respect to significant changes of energy parameters as well as increased hypothalamic expression of FXR target genes and importantly, we could observe that our dose was selective to FXR activation since FXR deficiency completely abolished the effect of ICV GW4064 on UCP1 and TH protein expression in BAT as well as the induction of FXR and CREB target genes in the ARH.

Our study provides the first demonstration that brain FXR is an important central regulator of energy homeostasis. Our findings pave the way for further investigations on the regulatory role of FXR in the communication between the brain and peripheral organs and the control of energy metabolism. Moreover, studies on the role of tonic activation of hypothalamic FXR by its endogenous BA ligands under physiological or pathological (e.g., cholestatitc) conditions are warranted. Finally, determination of the contribution of brain FXR activation to the clinical actions of synthetic FXR ligands, several of which being currently in development for the treatment of metabolic diseases, such as non-alcoholic fatty liver disease, will be of interest.

## Data Availability Statement

The raw data supporting the conclusions of this article will be made available by the authors, without undue reservation.

## Ethics Statement

The animal study was reviewed and approved by CEEA 75.

## Author Contributions

BDc, KB, and BS designed the research and wrote the manuscript. BDc, KB, DD, EN, CB, and J-SA performed the experiments. EC performed the metabolic cages experiments. ED and SD participated in animal experiments. JC and BDp performed the GW4064 assay. CM performed and analyzed the sympathetic nerve activity recording. SL and AT analyzed the results. All authors contributed to the article and approved the submitted version.

## Conflict of Interest

The authors declare that the research was conducted in the absence of any commercial or financial relationships that could be construed as a potential conflict of interest. The reviewer DV declared a shared affiliation, though no other collaboration, with several of the authors, KB, BDc, DD, CB, SD, EN, ED, EC, JC, EV, J-SA, AT, BS, BDp, SL.

## Publisher’s Note

All claims expressed in this article are solely those of the authors and do not necessarily represent those of their affiliated organizations, or those of the publisher, the editors and the reviewers. Any product that may be evaluated in this article, or claim that may be made by its manufacturer, is not guaranteed or endorsed by the publisher.

## Supplementary Material

Supplementary Figure 1 Effect of 6-days cerebral treatment with GW4064 on gene expression in BAT. mRNA expression of genes involved in glucose, lipid metabolism in BAT. The values are normalized to cyclophilin. Data are mean ± SEM. **P* < 0.05, ^**^*P* < 0.01, ^***^*P* < 0.001, Unpaired Student’s *t* test. Vehicle group is indicated as open bars, GW4064 group as black bars.

Supplementary Figure 2 Effect of 6-days cerebral treatment with GW4064 on gene expression of BAT in an 8 h-cold exposure paradigm. **(A–E)** *Adra1a*, *Adra2a*, *Adrb3*, *Pgc1a*, *Dio2* mRNA expression in BAT of mice placed at room temperature (23°C) or 4°C. The values are normalized to cyclophilin or 18 s. Data are mean ± SEM. *(VEH 23°C vs VEH 4°C) # (GW4064 23°C vs GW4064 4°C) *P* < 0.05, Two-Way ANOVA followed by Tukey *post hoc*. 23°C group is indicated as open bars, 4°C group as black bars.

Supplementary Figure 3 Effect of 6-days cerebral treatment with GW4064 on FXR target gene expression in liver. **(A,B)** *Shp* and *Bsep* mRNA expression in liver by q-PCR. The values are normalized to cyclophilin. Data are mean ± SEM. **P* < 0.05, ^**^*P* < 0.01, ^***^*P* < 0.001, Unpaired Student’s *t* test. Vehicle group is indicated as open bars, GW4064 group as black bars.

Supplementary Table 1 Primer for Sybr green and Taqman techniques.

## References

[B1] Abdelkader N. F. Safar M. M. Salem H. A. (2016). Ursodeoxycholic acid ameliorates apoptotic cascade in the rotenone model of Parkinson’s disease: modulation of mitochondrial perturbations. *Mol. Neurobiol.* 53 810–817. 10.1007/s12035-014-9043-8 25502462

[B2] Abdelkarim M. Caron S. Duhem C. Prawitt J. Dumont J. Lucas A. (2010). The Farnesoid X receptor regulates adipocyte differentiation and function by promoting peroxisome proliferator-activated receptor- and interfering with the Wnt/-catenin pathways. *J. Biol. Chem.* 285 36759–36767. 10.1074/jbc.M110.166231 20851881 PMC2978604

[B3] Bantubungi K. Prawitt J. Staels B. (2012). Control of metabolism by nutrient-regulated nuclear receptors acting in the brain. *J. Steroid Biochem. Mol. Biol.* 130 126–137. 10.1016/j.jsbmb.2011.10.002 22033286

[B4] Bell S. M. Barnes K. Clemmens H. Al-Rafiah A. R. Al-ofi E. A. Leech V. (2018). Ursodeoxycholic acid improves mitochondrial function and redistributes drp1 in fibroblasts from patients with either sporadic or familial Alzheimer’s disease. *J. Mol. Biol.* 430 3942–3953. 10.1016/j.jmb.2018.08.019 30171839 PMC6193139

[B5] Billinger M. Seiler C. Fleisch M. Eberli F. R. Meier B. Hess O. M. (2001). Do beta-adrenergic blocking agents increase coronary flow reserve? *J. Am. Coll. Cardiol.* 38 1866–1871. 10.1016/S0735-1097(01)01664-3 11738286

[B6] Cannon B. (2004). Brown adipose tissue: function and physiological significance. *Physiol. Rev.* 84 277–359. 10.1152/physrev.00015.2003 14715917

[B7] Cariou B. Bouchaert E. Abdelkarim M. Dumont J. Caron S. Fruchart J.-C. (2007). FXR-deficiency confers increased susceptibility to torpor. *FEBS Lett.* 581 5191–5198. 10.1016/j.febslet.2007.09.064 17950284

[B8] Castellanos-Jankiewicz A. Guzmán-Quevedo O. Fénelon V. S. Zizzari P. Quarta C. Bellocchio L. (2021). Hypothalamic bile acid-TGR5 signaling protects from obesity. *Cell Metab.* 33 1483–1492.e10. 10.1016/j.cmet.2021.04.009 33887197

[B9] Castro-Caldas M. Carvalho A. N. Rodrigues E. Henderson C. J. Wolf C. R. Rodrigues C. M. P. (2012). Tauroursodeoxycholic acid prevents MPTP-induced dopaminergic cell death in a mouse model of Parkinson’s disease. *Mol. Neurobiol.* 46 475–486. 10.1007/s12035-012-8295-4 22773138

[B10] Chávez-Talavera O. Tailleux A. Lefebvre P. Staels B. (2017). Bile acid control of metabolism and inflammation in obesity, Type 2 diabetes, dyslipidemia, and nonalcoholic fatty liver disease. *Gastroenterology* 152 1679–1694.e3. 10.1053/j.gastro.2017.01.055 28214524

[B11] Eggink H. M. Tambyrajah L. L. van den Berg R. Mol I. M. van den Heuvel J. K. Koehorst M. (2018). Chronic infusion of taurolithocholate into the brain increases fat oxidation in mice. *J. Endocrinol.* 236 85–97. 10.1530/JOE-17-0503 29233934

[B12] Ek L. Åblad B. (1971). Effects of three beta adrenergic receptor blockers on myocardial oxygen consumption in the dog. *Eur. J. Pharmacol.* 14 19–28. 10.1016/0014-2999(71)90118-X 4396658

[B13] Elia A. E. Lalli S. Monsurrò M. R. Sagnelli A. Taiello A. C. Reggiori B. (2016). Tauroursodeoxycholic acid in the treatment of patients with amyotrophic lateral sclerosis. *Eur. J. Neurol.* 23 45–52. 10.1111/ene.12664 25664595 PMC5024041

[B14] Gofflot F. Chartoire N. Vasseur L. Heikkinen S. Dembele D. Le Merrer J. (2007). Systematic gene expression mapping clusters nuclear receptors according to their function in the brain. *Cell* 131 405–418. 10.1016/j.cell.2007.09.012 17956739

[B15] Hall K. D. Heymsfield S. B. Kemnitz J. W. Klein S. Schoeller D. A. Speakman J. R. (2012). Energy balance and its components: implications for body weight regulation. *Am. J. Clin. Nutr.* 95 989–994. 10.3945/ajcn.112.036350 22434603 PMC3302369

[B16] Harms M. Seale P. (2013). Brown and beige fat: development, function and therapeutic potential. *Nat. Med.* 19 1252–1263. 10.1038/nm.3361 24100998

[B17] Herzig S. Long F. Jhala U. S. Hedrick S. Quinn R. Bauer A. (2001). CREB regulates hepatic gluconeogenesis through the coactivator PGC-1. *Nature* 413 179–183. 10.1038/35093131 11557984

[B18] Houssiere A. Gujic M. Deboeck G. Ciarka A. Naeije R. van de Borne P. (2007). Increased metaboreflex activity is related to exercise intolerance in heart transplant patients. *Am. J. Physiol. Heart Circ. Physiol.* 293 H3699–H3706. 10.1152/ajpheart.00694.2007 17921330

[B19] Huang C. Wang J. Hu W. Wang C. Lu X. Tong L. (2016). Identification of functional farnesoid X receptors in brain neurons. *FEBS Lett.* 590 3233–3242. 10.1002/1873-3468.12373 27545319

[B20] Huang F. Wang T. Lan Y. Yang L. Pan W. Zhu Y. (2015). Deletion of mouse FXR gene disturbs multiple neurotransmitter systems and alters neurobehavior. *Front. Behav. Neurosci.* 9:70. 10.3389/fnbeh.2015.00070 25870546 PMC4378301

[B21] Keene C. D. Rodrigues C. M. P. Eich T. Chhabra M. S. Steer C. J. Low W. C. (2002). Tauroursodeoxycholic acid, a bile acid, is neuroprotective in a transgenic animal model of Huntington’s disease. *Proc. Natl. Acad. Sci. U.S.A.* 99 10671–10676. 10.1073/pnas.162362299 12149470 PMC125009

[B22] Keene C. D. Rodrigues C. M. P. Eich T. Linehan-Stieers C. Abt A. Kren B. T. (2001). A bile acid protects against motor and cognitive deficits and reduces striatal degeneration in the 3-nitropropionic acid model of Huntington’s disease. *Exp. Neurol.* 171 351–360. 10.1006/exnr.2001.7755 11573988

[B23] Kim K.-S. Seeley R. J. Sandoval D. A. (2018). Signalling from the periphery to the brain that regulates energy homeostasis. *Nat. Rev. Neurosci.* 19 185–196. 10.1038/nrn.2018.8 29467468 PMC9190118

[B24] Lang S. A. Maron M. B. Signs S. A. (1989). Oxygen consumption after massive sympathetic nervous system discharge. *Am. J. Physiol. Endocrinol. Metab.* 256 E345–E351. 10.1152/ajpendo.1989.256.3.E345 2923204

[B25] Lefebvre P. Cariou B. Lien F. Kuipers F. Staels B. (2009). Role of bile acids and bile acid receptors in metabolic regulation. *Physiol. Rev.* 89 147–191. 10.1152/physrev.00010.2008 19126757

[B26] Lo A. C. Callaerts-Vegh Z. Nunes A. F. Rodrigues C. M. P. D’Hooge R. (2013). Tauroursodeoxycholic acid (TUDCA) supplementation prevents cognitive impairment and amyloid deposition in APP/PS1 mice. *Neurobiol. Dis.* 50 21–29. 10.1016/j.nbd.2012.09.003 22974733

[B27] Maloney P. R. Parks D. J. Haffner C. D. Fivush A. M. Chandra G. Plunket K. D. (2000). Identification of a chemical tool for the orphan nuclear receptor FXR. *J. Med. Chem.* 43 2971–2974. 10.1021/jm0002127 10956205

[B28] McMillin M. Grant S. Frampton G. Petrescu A. D. Kain J. Williams E. (2018). FXR-mediated cortical cholesterol accumulation contributes to the pathogenesis of type A hepatic encephalopathy. *Cell. Mol. Gastroenterol. Hepatol.* 6 47–63. 10.1016/j.jcmgh.2018.02.008 29928671 PMC6008252

[B29] Moreira S. Fonseca I. Nunes M. J. Rosa A. Lemos L. Rodrigues E. (2017). Nrf2 activation by tauroursodeoxycholic acid in experimental models of Parkinson’s disease. *Exp. Neurol.* 295 77–87. 10.1016/j.expneurol.2017.05.009 28552716

[B30] Narkiewicz K. van de Borne P. Montano N. Hering D. Kara T. Somers V. K. (2006). Sympathetic neural outflow and chemoreflex sensitivity are related to spontaneous breathing rate in normal men. *Hypertension* 47 51–55. 10.1161/01.HYP.0000197613.47649.02 16344363

[B31] Pandey S. C. (2003). Anxiety and alcohol abuse disorders: a common role for CREB and its target, the neuropeptide Y gene. *Trends Pharmacol. Sci.* 24 456–460. 10.1016/S0165-6147(03)00226-8 12967770

[B32] Piech-Dumas K. M. Tank A. W. (1999). CREB mediates the cAMP-responsiveness of the tyrosine hydroxylase gene: use of an antisense RNA strategy to produce CREB-deficient PC12 cell lines. *Mol. Brain Res.* 70 219–230. 10.1016/S0169-328X(99)00149-7 10407170

[B33] Popescu I. R. Helleboid-Chapman A. Lucas A. Vandewalle B. Dumont J. Bouchaert E. (2010). The nuclear receptor FXR is expressed in pancreatic beta-cells and protects human islets from lipotoxicity. *FEBS Lett.* 584 2845–2851. 10.1016/j.febslet.2010.04.068 20447400

[B34] Prawitt J. Abdelkarim M. Stroeve J. H. M. Popescu I. Duez H. Velagapudi V. R. (2011). Farnesoid X receptor deficiency improves glucose homeostasis in mouse models of obesity. *Diabetes Metab. Res. Rev.* 60 1861–1871. 10.2337/db11-0030 21593203 PMC3121443

[B35] Razzoli M. Frontini A. Gurney A. Mondini E. Cubuk C. Katz L. S. (2016). Stress-induced activation of brown adipose tissue prevents obesity in conditions of low adaptive thermogenesis. *Mol. Metab.* 5 19–33. 10.1016/j.molmet.2015.10.005 26844204 PMC4703853

[B36] Rosa A. I. Duarte-Silva S. Silva-Fernandes A. Nunes M. J. Carvalho A. N. Rodrigues E. (2018). Tauroursodeoxycholic acid improves motor symptoms in a mouse model of Parkinson’s disease. *Mol. Neurobiol.* 55 9139–9155. 10.1007/s12035-018-1062-4 29651747

[B37] Sánchez-Lasheras C. Christine Könner A. Brüning J. C. (2010). Integrative neurobiology of energy homeostasis-neurocircuits, signals and mediators. *Front. Neuroendocrinol.* 31 4–15. 10.1016/j.yfrne.2009.08.002 19729032

[B38] Seok S. Fu T. Choi S.-E. Li Y. Zhu R. Kumar S. (2014). Transcriptional regulation of autophagy by an FXR–CREB axis. *Nature* 516 108–111. 10.1038/nature13949 25383523 PMC4257899

[B39] Seyer P. Vallois D. Poitry-Yamate C. Schütz F. Metref S. Tarussio D. (2013). Hepatic glucose sensing is required to preserve β cell glucose competence. *J. Clin. Invest.* 123 1662–1676. 10.1172/JCI65538 23549084 PMC3613916

[B40] Shimizu I. Aprahamian T. Kikuchi R. Shimizu A. Papanicolaou K. N. MacLauchlan S. (2014). Vascular rarefaction mediates whitening of brown fat in obesity. *J. Clin. Invest.* 124 2099–2112. 10.1172/JCI71643 24713652 PMC4001539

[B41] Sinal C. J. Tohkin M. Miyata M. Ward J. M. Lambert G. Gonzalez F. J. (2000). Targeted disruption of the nuclear receptor FXR/BAR impairs bile acid and lipid homeostasis. *Cell* 102 731–744. 10.1016/S0092-8674(00)00062-3 11030617

[B42] Swanson L. W. Sawchenko P. E. (1983). Hypothalamic integration: organization of the paraventricular and supraoptic nuclei. *Annu. Rev. Neurosci.* 6 269–324. 10.1146/annurev.ne.06.030183.001413 6132586

[B43] Trabelsi M.-S. Daoudi M. Prawitt J. Ducastel S. Touche V. Sayin S. I. (2015). Farnesoid X receptor inhibits glucagon-like peptide-1 production by enteroendocrine L cells. *Nat. Commun.* 6:7629. 10.1038/ncomms8629 26134028 PMC4579574

[B44] Tully D. C. Rucker P. V. Chianelli D. Williams J. Vidal A. Alper P. B. (2017). Discovery of Tropifexor (LJN452), a Highly Potent Non-bile Acid FXR Agonist for the Treatment of Cholestatic Liver Diseases and Nonalcoholic Steatohepatitis (NASH). *J. Med. Chem.* 60 9960–9973. 10.1021/acs.jmedchem.7b00907 29148806

[B45] Vaz A. R. Cunha C. Gomes C. Schmucki N. Barbosa M. Brites D. (2015). Glycoursodeoxycholic acid reduces matrix metalloproteinase-9 and Caspase-9 activation in a cellular model of superoxide dismutase-1 neurodegeneration. *Mol. Neurobiol.* 51 864–877. 10.1007/s12035-014-8731-8 24848512

[B46] Watanabe M. Horai Y. Houten S. M. Morimoto K. Sugizaki T. Arita E. (2011). Lowering bile acid pool size with a synthetic farnesoid X Receptor (FXR) agonist induces obesity and diabetes through reduced energy expenditure. *J. Biol. Chem.* 286 26913–26920. 10.1074/jbc.M111.248203 21632533 PMC3143650

[B47] Watanabe M. Houten S. M. Mataki C. Christoffolete M. A. Kim B. W. Sato H. (2006). Bile acids induce energy expenditure by promoting intracellular thyroid hormone activation. *Nature* 439 484–489. 10.1038/nature04330 16400329

[B48] Waterson M. J. Horvath T. L. (2015). Neuronal regulation of energy homeostasis: beyond the hypothalamus and feeding. *Cell Metab.* 22 962–970. 10.1016/j.cmet.2015.09.026 26603190

[B49] Witte K. K. A. (2003). The effects of and blockade on ventilatory responses to exercise in chronic heart failure. *Heart* 89 1169–1173. 10.1136/heart.89.10.1169 12975409 PMC1767894

[B50] Witte K. K. A. Notarius C. F. Ivanov J. Floras J. S. (2008). Muscle sympathetic nerve activity and ventilation during exercise in subjects with and without chronic heart failure. *Can. J. Cardiol.* 24 275–278. 10.1016/S0828-282X(08)70176-4 18401467 PMC2644031

[B51] Yang L. McKnight G. S. (2015). Hypothalamic PKA regulates leptin sensitivity and adiposity. *Nat. Commun.* 6:8237. 10.1038/ncomms9237 26381935 PMC4576457

[B52] Zhang W. Bi S. (2015). Hypothalamic regulation of brown adipose tissue thermogenesis and energy homeostasis. *Front. Endocrinol.* 6:136. 10.3389/fendo.2015.00136 26379628 PMC4553396

[B53] Zhang Y. Ge X. Heemstra L. A. Chen W.-D. Xu J. Smith J. L. (2012). Loss of FXR protects against diet-induced obesity and accelerates liver carcinogenesis in ob/ob Mice. *Mol. Endocrinol.* 26 272–280. 10.1210/me.2011-1157 22261820 PMC3275160

