# Supplementary figures and images for: Farnesoid X Receptor Activation in Brain Alters Brown Adipose Tissue Function via the Sympathetic System
Source: Front Mol Neurosci. 2022 Jan 4;14:808603. doi: 10.3389/fnmol.2021.808603 (PMC8764415; doi:10.3389/fnmol.2021.808603)

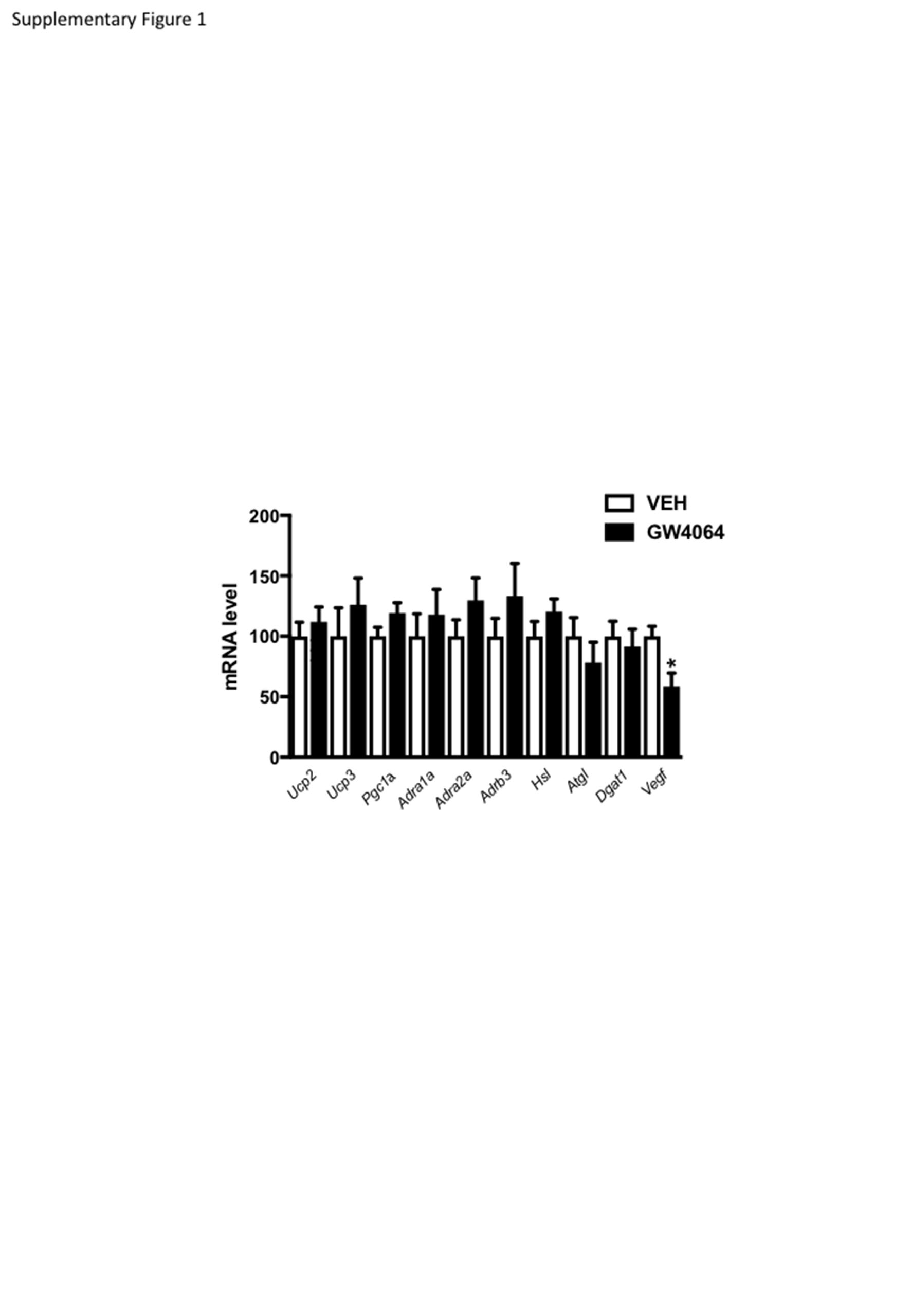

Supplement: Supplementary Figure 1 — Effect of 6-days cerebral treatment with GW4064 on gene expression in BAT. mRNA expression of genes involved in glucose, lipid metabolism in BAT. The values are normalized to cyclophilin. Data are mean ± SEM. *P < 0.05, **P < 0.01, ***P < 0.001, Unpaired Student’s t test. Vehicle group is indicated as open bars, GW4064 group as black bars. [file Image_1.tiff]

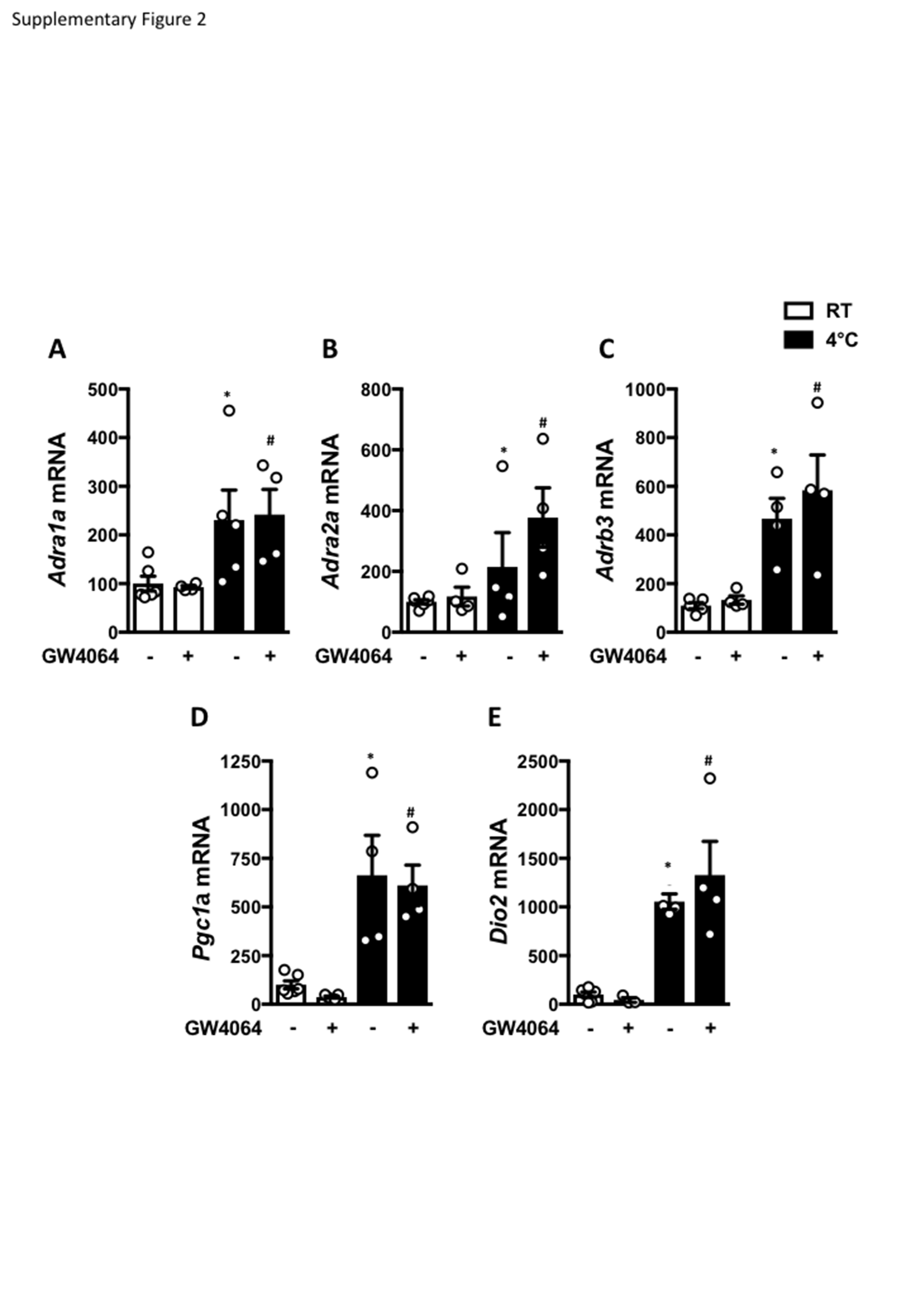

Supplement: Supplementary Figure 2 — Effect of 6-days cerebral treatment with GW4064 on gene expression of BAT in an 8 h-cold exposure paradigm. (A–E) Adra1a, Adra2a, Adrb3, Pgc1a, Dio2 mRNA expression in BAT of mice placed at room temperature (23°C) or 4°C. The values are normalized to cyclophilin or 18 s. Data are mean ± SEM. *(VEH 23°C vs VEH 4°C) # (GW4064 23°C vs GW4064 4°C) P < 0.05, Two-Way ANOVA followed by Tukey post hoc. 23°C group is indicated as open bars, 4°C group as black bars. [file Image_2.tiff]

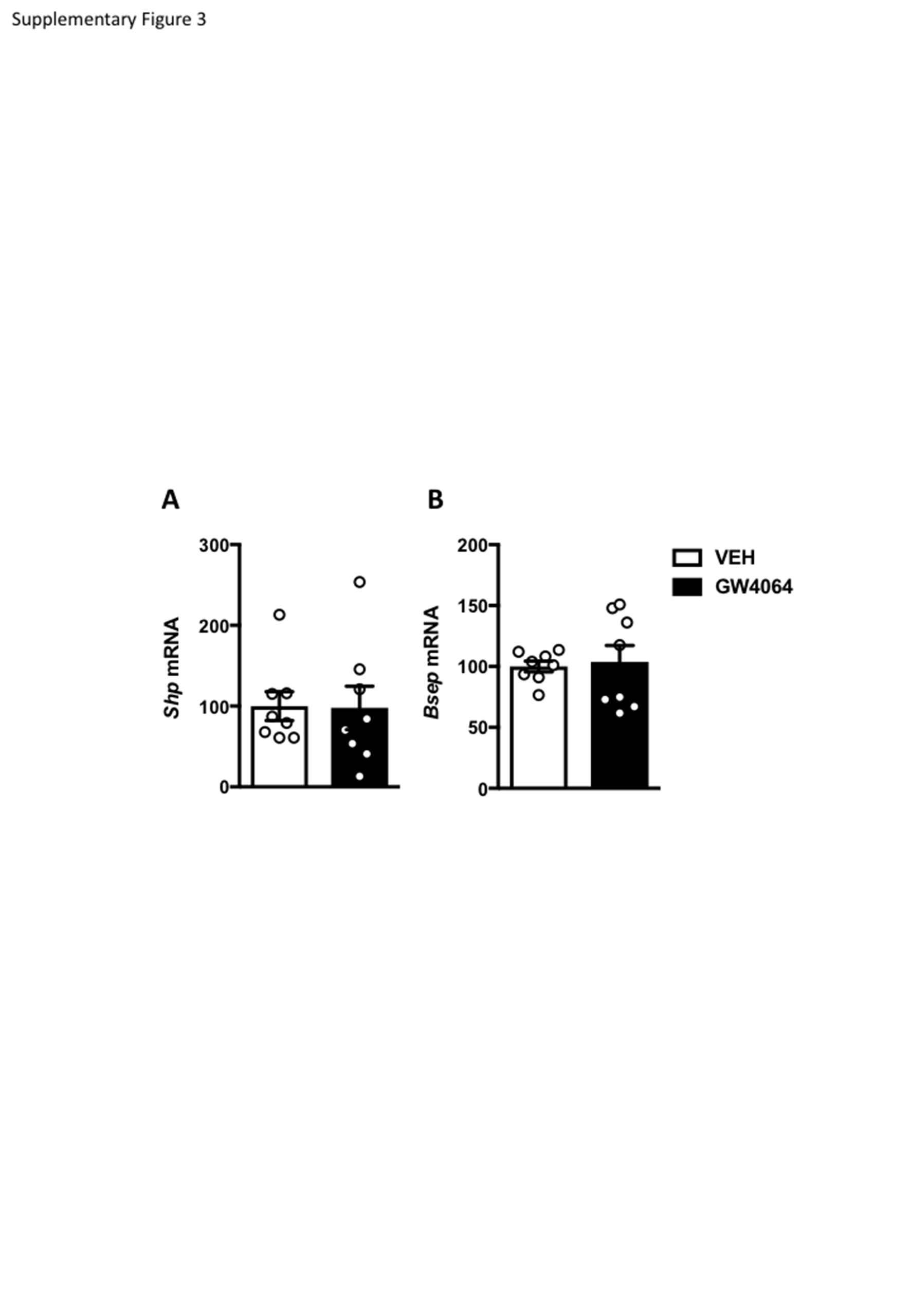

Supplement: Supplementary Figure 3 — Effect of 6-days cerebral treatment with GW4064 on FXR target gene expression in liver. (A,B) Shp and Bsep mRNA expression in liver by q-PCR. The values are normalized to cyclophilin. Data are mean ± SEM. *P < 0.05, **P < 0.01, ***P < 0.001, Unpaired Student’s t test. Vehicle group is indicated as open bars, GW4064 group as black bars. [file Image_3.tiff]
