# Supplementary material for: Farnesoid X Receptor Activation in Brain Alters Brown Adipose Tissue Function via the Sympathetic System
Source: Front Mol Neurosci. 2022 Jan 4;14:808603. doi: 10.3389/fnmol.2021.808603 (PMC8764415; doi:10.3389/fnmol.2021.808603)
Supplement: Supplementary Table 1 — Primer for Sybr green and Taqman techniques. [file Table_1.pdf]

Supplementary Methods

Primer for Sybr green and Taqman techniques

Gene	NCBI number	Sequences 5'-3'	
		Forward	*Reverse*
Cyclophilin	NM_001123068	GCATACGGGTCCTGGCATCTTGTCC	ATGGTGATCTTC TTGCTGGTCTTGC
FXR (α1-2)	NM_001163504.1	ATGAATCTGATTGGGCACTCCCAT	AAATAAGCTTTCAGAAAGAGAAAA
SHP	NM_011850	ACGATCCTCTTCAACCCAGA	AGGGTAGAGGCCATGAGGAG
BSEP	NM_0210022	GTCTGACTCAGTGATTCTTCGC	GAGCAATGCGCACACACTTC
NPY	NM_023456,2	ACTCCGCTCTGCGACACTAC	TCTCAGGGCTGGATCTCTTG
PGC1a	NM_008904	CTACAGACACCGCACACACC	TCATCCCTCTTGAGCCTTTC
HSL	NM_010719.5	GTGGCGAAAAGGCAAGATCAA	CTGGCTTGAGAAGAAGGCCA
ATGL	NM_025802.3	CCACATTGGCGTGGCCTCCT	AACCGCTTCCGGGCCTCCTT
DGAT1	NM_010046.3	GACGGCTACTGGGATCTGA	TCACCACACACCAATTCAGG
UCP1	NM_009463	GGAGGTGTGGCAGTGTTCATTGG	AGCATTGTAGGTCCCCGTGTAGCG
UCP2	NM_011671.5	AGCCCAGCCTACAGATGTGGTAAA	TATCCAGTGGTCGAGTCGTGTCAA
UCP3	AF 030164	CCTCCATTCGAATTGGCCTC	CCCTCTGTATTTCCTCTCTCCTCC
VEGF	NM_001025250.2	AGCACAGCAGATGTGAATGC	AATGCTTTCTCCGCTCTGAA
DIO2	NM_010050.2	TCGCGGAGAGTGGAGGCGCA	CGTGCACCACACTGGAATTGGGAGC
Gene	NCBI number		Taqman assay ID
TH	NM_009377.1		Mm00447557_m1
Adrb3	NM_013462.3		Mm02601819_g1
Adra1a	NM_001271759.1, NM_001271760.1, NM_013461.4		Mm00442668_m1
Adra2a	NM_007417.4		Mm00845383_s1
CRTC2	NM_028881.2		Mm01219960_m1
18s	TaqMan Ribosomal RNA Control Reagents (Thermofischer, 4308329)		

FXR : Farnesoid X Receptor ; SHP : Small Heterodimer Partner ; BESP : Bile Salt Export Pump ; NPY : Neuropeptide Y ; PGC1a : Peroxisome proliferator-activated receptor Gamma Coactivator 1-alpha ; HSL : Hormone-Sensitive Lipase ; ATGL : Adipose Triglyceride Lipase ; DGAT1 : Diacylglycerol O-Acyltransferase 1 : UCP1 : Uncoupling protein1 ; UCP2 : Uncoupling protein2 ; UCP3 : Uncoupling protein3 ; VEGF : Vascular Endothelial Growth Factor ; DIO2 : Iodothyronine Deiodinase 2 ; TH : Tyrosine hydroxylase ; Adrb3 : β3-adrenergic receptor ; Adra1a : α1-adrenergic receptor ; Adra2a : α2-adrenergic receptor ; CRTC2 : CREB Regulated Transcription Coactivator 2.
